# Phytocannabinoids Reduce Seizures in Larval Zebrafish and Affect Endocannabinoid Gene Expression

**DOI:** 10.3390/biom13091398

**Published:** 2023-09-16

**Authors:** Roshni Kollipara, Evan Langille, Cameron Tobin, Curtis R. French

**Affiliations:** 1 Department of Biomedical Sciences, Faculty of Medicine, Memorial University of Newfoundland, St. John’s, NL A1B 3V6, Canada; rskk10@mun.ca (R.K.); ctobin19@mun.ca (C.T.); 2 Department of Chemistry, Faculty of Science, Memorial University of Newfoundland, St. John’s, NL A1B 3X7, Canada; ealangille@mun.ca

**Keywords:** cannabis, zebrafish, phytocannabinoid, endocannabinoid, seizure, epilepsy, GPR55

## Abstract

Cannabis has demonstrated anticonvulsant properties, and about thirty percent of epileptic patients do not have satisfactory seizure management with standard treatment and could potentially benefit from cannabis-based intervention. Here, we report the use of cannabinoids to treat pentylenetetrazol (PTZ)-induced convulsions in a zebrafish model, their effect on gene expression, and a simple assay for assessing their uptake in zebrafish tissues. Using an optimized behavioral assay, we show that cannabidiol (CBD) and cannabichromene (CBC) and cannabinol (CBN) are effective at reducing seizures at low doses, with little evidence of sedation, and our novel HPLC assay indicates that CBC is effective with the lowest accumulation in larval tissues. All cannabinoids tested were effective at higher concentrations. Pharmacological manipulation of potential receptors demonstrates that Gpr55 partially mediates the anticonvulsant effects of CBD. Treatment of zebrafish larvae with endocannabinoids, such as 2-arachidonoylglycerol (2-AG) and anandamide (AEA), altered larvae movement, and the expression of genes that regulate their metabolism was affected by phytocannabinoid treatment, highlighting the possibility that changes to endocannabinoid levels may represent one facet of the anticonvulsant effect of phytocannabinoids.

## 1. Introduction

Epilepsy is a complex disorder characterized by repeated seizures, which are rapid discharges of action potential in the brain that can result in uncontrolled movement and periods of lack of awareness. Seizures, along with additional disease manifestations, such as loss of senses, disturbances to the autonomic nervous system, and emotional changes, can profoundly affect the quality of life. Epilepsy has diverse etiologies, confounded by several types of seizures and six categories of epilepsy as per the ILAE guidelines [1]. Clinical seizure control can be difficult to achieve because approximately one-third of epilepsies do not have a known cause, and greater than 30% of patients with epilepsy do not respond to currently available treatments (refractory epilepsy) [2,3]. Additionally, adverse effects to current anti-seizure medications (ASMs), such as drowsiness, gastrointestinal distress, aseptic meningitis, and risk to the unborn, often result in discontinued treatment [4]. Furthermore, treatment with standard ASMs can induce cognitive dysfunction in patients and animal models, highlighting the need for additional ASM discovery and validation [5,6].

Animal models play a crucial role in epilepsy research, and some of the original ASMs were discovered after studies on animal models, as discussed by Perucca, 2019 [7]. The non-competitive GABA_A_ receptor antagonist pentylenetetrazole (PTZ), for example, invokes concentration-dependent seizure-like behavior in rodents and zebrafish [8,9,10,11]. Zebrafish seizure models are becoming more prominent in ASM candidate studies due to their high fecundity, low cost to maintain, and stereotypical behaviors that beget a high-throughput workflow. Zebrafish PTZ models display stereotypical convulsions and increased brain activity, as quantified through calcium imaging, and respond to treatment with approved and candidate ASMs [12,13,14,15,16].

Cannabis is a versatile medicinal plant that has been used to treat convulsions since ancient times [17,18]. There are over 100 phytocannabinoids found naturally in cannabis [19,20,21]. In this work, we focus on six of the most prevalent phytocannabinoids—cannabidiol (CBD), Δ^9^-tetrahydrocannabinol (Δ^9^-THC), Δ^8^-tetrahydrocannabinol (Δ^8^-THC), cannabinol (CBN), cannabichromene (CBC), and cannabigerol (CBG)—and their role in reducing hyperactivity in an optimized zebrafish movement assay. CBD is the active component in Epidiolex^®^, which the Federal Food and Drug Administration (FDA) and European Medicines Agency (EMA) have approved for treating patients 2 years and older with severe genetic childhood convulsant syndromes, such as Dravet Syndrome and Lennox–Gastaut Syndrome [22,23,24]. Emerging evidence from animal models indicates that additional cannabinoids may also have anticonvulsant effects. CBN has been reported to display anticonvulsant effects similar to that of CBD in a zebrafish model of Dravet syndrome [25]. CBC is one of the most abundant cannabinoids in cannabis but has not been well studied with respect to epilepsy, yet is known to exhibit neuroprotective effects in cultured mouse neural cells [26]. There is also evidence that CBC, when used in combination with CBD or Δ^9^-THC, can help alleviate symptoms of insomnia and depression in humans and rats [27,28]. CBG was determined to be non-psychotropic and is of interest commercially as it is abundant in some commonly used hemp plants, but little is known regarding its potential anticonvulsant properties [29].

Treatment with Δ^9^-THC in combination with CBD has been the main focus of recent studies and has been associated with improved symptoms of neurodegenerative diseases, pain perception, and depression, as reviewed by Zhang and colleagues in 2022 [30]. A combination of Δ^9^-THC and CBD has also been shown to be anticonvulsant in a mouse model of Dravet Syndrome and zebrafish models of neuro-hyperactivity [31,32]. Sole treatment with Δ^9^-THC is generally reserved for extreme medical cases because of the psychoactive effects and can be used to treat severe emesis, as shown in pilot clinical studies [33]. There is also evidence that Δ^9^-THC treatment inhibits fear learning in a zebrafish model [34], and it is being investigated for the treatment of psychological illnesses, such as post-traumatic stress disorder (PTSD), in humans [35]. Δ^8^-THC is a less psychoactive analog of Δ^9^-THC, and a self-reporting study on the effects of Δ^8^-THC indicated a more tolerable side effect profile while maintaining pain relief and relaxation as compared to Δ^9^-THC [36]. There is significant value in understanding the effects of phytocannabinoids and their interactions with each other, particularly in the case of epilepsy, as it has been observed in patients, as well as in zebrafish models, that cannabis extracts have different anticonvulsant efficacy even when normalized for the dose of CBD [19].

The mechanism by which CBD and other cannabinoids elicit their anticonvulsant properties is not well understood. While interaction with receptors of the endocannabinoid signaling system has been well documented [37], it is not clear which receptors are required to transduce the anticonvulsant effects of phytocannabinoids. A number of receptors have been proposed, including the G-protein-coupled cannabinoid receptors CBR1 and CBR2 and G-protein orphan receptors, such a GPR18 and GPR55, as well as transient receptor potential cation channel receptors, such as TRPV1 [38,39,40,41,42]. It is also not clear whether signaling induced by phytocannabinoids affect the synthesis of natural endocannabinoid ligands, such as anandamide (AEA) and 2-arachidonoylglycerol (2-AG), through feedback mechanisms. Activation of CBR1 and CBR2 receptors via these endocannabinoids can inhibit neurotransmitter release [43], and changes in 2-AG levels have been noted during seizures [44] and potentially provide neuroprotective effects [45]. Changes in endocannabinoid synthesis could, thus, represent a component of the anticonvulsant mechanism of phytocannabinoids. There are also genetic markers of seizures that may be affected by the introduction of phytocannabinoids that may help to elucidate their mode of action [8,12]. For example, the expression of *peptide YY (pyya)*, *v-fos FBJ murine osteosarcoma viral oncogene homolog Ab (fosab)*, and *brain-derived neurotrophic factor (bdnf)* is altered during PTZ-induced seizures in animal models [8,12,46,47], yet it is unknown if phytocannabinoids may affect their expression as part of their therapeutic potential.

In this work, we test the use of six common phytocannabinoids and two known endocannabinoids for their ability to reduce seizures in zebrafish using behavioral tracking and gene expression readouts. Utilizing a novel HPLC-UV-based assay to quantify phytocannabinoid levels in zebrafish tissues after seizure activity, we demonstrate that CBC works at lower effective doses than CBD, without evidence of adverse side effects or sedation. Notably, we demonstrate a clear role for the orphan receptor Gpr55 in mediating the anticonvulsant effects of CBD in the zebrafish PTZ model. Incubation of larvae in the endocannabinoid 2-AG affected larval movement and treatment with phytocannabinoids altered the expression of key genes involved in the metabolism of endocannabinoids, indicating that an effect on endocannabinoid signaling may be a key component of seizure rescue via phytocannabinoid treatment in the PTZ model.

## 2. Materials and Methods

### 2.1. Zebrafish Husbandry

Wild-type zebrafish (strain AB) were reared and staged under standard conditions, as previously described [48]. All experiments were completed in accordance with Memorial University of Newfoundland’s Animal Care Committee (Approval #20222627) and the Canadian Council on Animal Care. Zebrafish larvae were reared in E3 embryo media at 28.5 degrees with a standard light cycle.

### 2.2. Cannabinoid Dosing and Seizure Tracking

All phytocannabinoids and PTZ were purchased from Sigma Aldrich (Oakville, ON, Canada). AEA, 2-AG and receptor antagonists were purchased from Cayman Chemicals (Ann Arbor, MI, USA). Larvae were collected and reared according to breeding pairs. One larva (6 dpf) was added per well in a 96-well plate, and 180 µL of cannabinoid or vehicle-treated embryo media was added. The plate was incubated at 28.5 °C for 30 min and then recorded in the dark, using an infrared camera in a tracking apparatus for 30 min with a 5 min acclimation. This provided baseline movement data (vehicle treated) and the level of sedation or hyperactivity induced by phytocannabinoid treatment. In the dark, PTZ was then added to a final concentration of 2.75 mM, and the plate was recorded for 30 additional minutes after a 5 min acclimation. Activity, measured as percent pixel change, was measured for 30 min using Noldus EthoVision XT 15 software. For receptor inhibition studies, receptor antagonists were added with phytocannabinoids to a final concentration of 2.5 μM (ML-193 and PSB-CB5) and 1 μM (AM251).

### 2.3. Phytocannabinoid Extraction

Larvae were pooled in groups of 12, according to the treatment received, rinsed twice in ice-cold embryo media, and euthanized by incubation on ice. Excess embryo media was removed and samples stored at −80 °C until extraction. All steps of extraction were completed at 4–8 °C. A total of 200 µL of extraction solvent (methanol with 0.1% formic acid) was added to the larvae, which were then bead beaten for 3 min in an equal volume of 1 mm glass beads. Homogenate was then centrifuged for 30 min at 18,000× *g* and the supernatant 0.2 µm filtered prior to HPLC analysis.

### 2.4. HPLC Analysis of Phytocannabinoids

Analytical HPLC was carried out on an HP 1050 system equipped with two HALO 2.7 µm C18 100 × 4.6 mm columns (200 mm total length). A diode array detector monitored 200–400 nm. Extracted wavelength chromatograms at 210 nm for CBG, CBD, Δ^9^-THC, and Δ^8^-THC; 222 nm for CBN; and 230 nm for CBC were used for quantification using TargetLynx version 4.1 software (Waters Corp., Milford, MA, USA) and can be found in Figure S1. The mobile phase consisted of an isocratic mixture of 17.5:82.5 water 0.1% formic acid: acetonitrile 0.1% formic acid, *v*/*v* at a flow rate of 1 mL min^−1^, resulting in a system backpressure of approximately 20 MPa. Triplicate 20 µL injections were performed per sample. External standards of the phytocannabinoids in extraction solvent were used to generate calibration curves (0, 0.1, 0.25, 0.5, 1, 2.5, 5, and 10 µg/mL), found in Figure S2, with analytical figures of merit presented in Table S1.

### 2.5. RT-qPCR

Larvae (6 dpf) were treated using the same method described for seizure tracking and pooled in groups of 12 for RNA isolation. RNA was isolated using the PureLink^TM^ RNA Mini Kit (Invitrogen, Waltham, MA, USA) with on-column DNAase treatment using the PureLink^TM^ DNase Set (Invitrogen), and cDNA synthesized using the High-Capacity cDNA Reverse Transcription Kit (ThermoFisher). qPCR was performed with Taqman Gene Expression Master Mix and Taqman assays (FAM) for *fosab* (Dr03100809_g1), *pyya* (Dr03138152_m1), *gde1* (Dr03434842_m1), *napepld* (Dr03117925_m1), *faah* (Dr03093136_m1), *ptgs2a* (Dr03080325_m1), and *ptgs1* (Dr03087197_m1) using a ViiA 7 qPCR machine. Wells were duplexed with the endogenous control, TATA-box binding protein (*tbp*) (VIC). Data were analyzed using the ΔΔCT method and presented as means ± standard error of the mean. Two biological replicates of 12 pooled larvae, with three technical replicates for each sample, were analyzed.

### 2.6. Statistics

Movement data were repeated three times, tended to be skewed, and differed significantly from normal distribution as assessed using a Kolmogorov–Smirnov Test. Significance was calculated using a Kruskal–Wallis non-parametric test with Dunn’s post hoc analysis. Statistical outliers were identified using Grubbs’ test and removed from analysis. All qPCR data were normally distributed, and significance was thus tested using a one-factor ANOVA with the Tukey post hoc test. For assessing phytocannabinoid movement effects, at least 67 larvae were used. For the receptor analysis, at least 34 larvae were used, while the analysis of endocannabinoids used a minimum of 40 larvae. Data were deemed statistically significant at the *p* < 0.05 level.

## 3. Results

### 3.1. Exposure to Phytocannabinoids Affects Baseline Larval Movement

**Figure 1:**
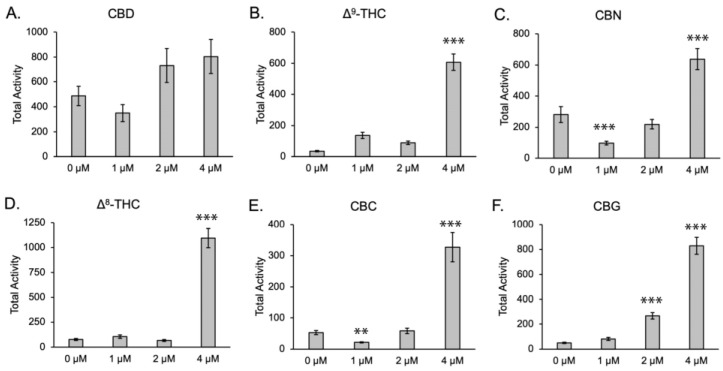
Analysis of movement in 6 dpf larvae exposed to phytocannabinoids and vehicle control (methanol). (**A**–**F**) Baseline activity of 6 phytocannabinoids. The “0 μM” indicates incubation in methanol only. Data presented as mean +/− SEM. ** *p* < 0.01, *** *p* < 0.001 (compared to 0 μM). n = 67–72 per condition.

Before the analysis of potential anticonvulsant activity, we assessed the effect of phytocannabinoid treatment on baseline larval movement. Activity was measured as total activity via pixel change. Analysis of phytocannabinoids in larvae without PTZ showed little evidence of sedation, defined as movement less than control larvae treated with a vehicle control (Figure 1). Doses of 1 μM and 2 μM of CBD, Δ^8^-THC, and Δ^9^-THC showed no statistical change from the vehicle control (Figure 1A,B,D). CBC and CBN showed evidence of sedation at the 1 μM dose, as movement was statistically less than that with vehicle controls, but not at 2 μM (Figure 1C,E). CBG significantly increased larval movement at the 2 and 4 μM doses (Figure 1F), as did all phytocannabinoids tested at the 4 μM dose.

### 3.2. CBD, CBC, and CBN Reduce PTZ-Induced Hyperactivity at Low Concentrations

**Figure 2:**
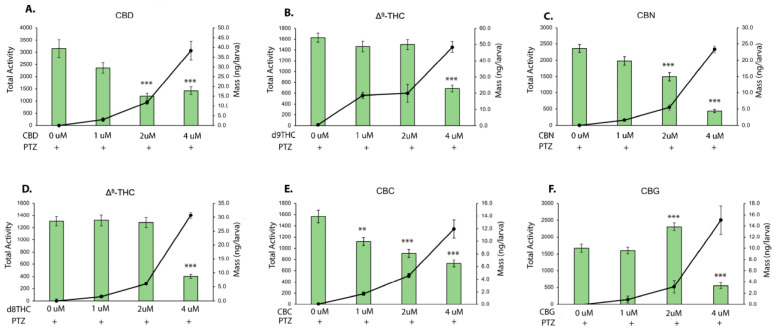
Analysis of PTZ-induced hyperactivity after pre-treatment with phytocannabinoids. (**A**–**F**) Total activity after PTZ and pretreatment with three doses (1, 2, and 4 μM, green bars) of 6 phytocannabinoids. The solid black line indicates the mass of cannabinoid isolated through HPLC analysis. Data graphed as mean +/− SEM, ** *p*< 0.01, *** *p* < 0.001 (movement data compared to 0 μM). n = 67–72 per treatment.

After recording movement in phytocannabinoids or a vehicle control, 2.75 mM PTZ was added to determine if preincubation with cannabinoids affected the ability to induce hyperactivity as a surrogate measure of seizures. Larvae were then analyzed for phytocannabinoid tissue accumulation using HPLC analysis. CBC inhibited PTZ-induced hyperactivity in a dose-dependent manner, with 1, 2, and 4 μM doses being statistically significant (Figure 2E). CBD and CBN reduced seizure activity, with 2 and 4 μM doses being statistically significant (Figure 2A,C). Both Δ^9^-THC and Δ^8^-THC only showed reduced movement at the 4 μM dose (Figure 2B,D), with movement being at or below the levels tested without PTZ (Figure 1B,D). Preincubation in CBG has a bimodal effect, with 2 μM resulting in increased activity compared to treatment with PTZ, and 4 μM resulting in less movement (Figure 2F).

Immediately after recording, larvae were used for phytocannabinoid quantification. All larvae displayed increased accumulation of cannabinoids with an increasing dose added to the water, with Δ^9^-THC accumulating at the highest levels (Figure 2, solid black line). Of note, CBC accumulated at the lowest levels in larvae, yet reduced hyperactivity at levels as low as 1.7 ng per larvae via the 1 μM dose added to fish water (Figure 2E).

### 3.3. Inhibition of Gpr55, but Not Cbr1 or Gpr18, Affects the Ability of CBD to Reduce PTZ-Induced Hyperactivity

**Figure 3:**
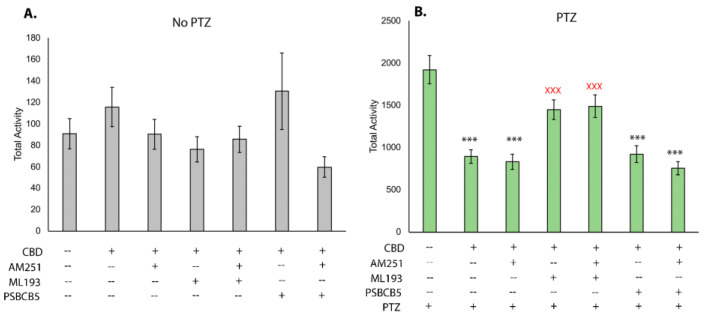
Inhibition of endocannabinoid receptors in zebrafish PTZ hyperactivity assay. Movement tracking after addition of CBD (2 μM) and receptor antagonists demonstrating the effect on baseline movement (**A**) all data not significant from vehicle controls) and after the addition of PTZ (2.75 mM (**B**). Final concentrations of receptor antagonists were 2.5 μM (ML-193 and PSB-CB5) and 1 μM AM251. Data graphed as mean +/− SEM. n = 34–36 larvae for each condition. *** statistically significant *p* < 0.001 when compared to PTZ only. Red X (XXX) indicates statistical significance from the combined PTZ and CBD treatment *p* < 0.001.

We next sought to determine which receptors may be necessary for CBD to reduce seizures and hyperactivity. Using well-established chemical antagonists of endocannabinoid receptors, we assessed hyperactivity and the robust reduction provided by CBD when endocannabinoid receptors are inhibited. Testing the baseline movement after addition of the Cbr1 antagonist AM251, the Gpr55 antagonist ML-193, and the Gpr18 antagonist PSB-CB5 demonstrates that none of the compounds in combination with CBD affects baseline movement (Figure 3A). After the addition of PTZ, CBD was able to reduce overall movement, as shown previously (Figure 2A and Figure 3B); however, when it was used in combination with Ml-193, movement was increased (Figure 3B). This level of movement was statistically higher than with CBD alone and not statistically different from PTZ-based movement, indicating that Gpr55 is required for the ability of CBD to reduce PTZ-induced hyperactivity. CBD was capable of reducing hyperactivity when larvae were treated with AM251 and PSB-CB5, indicating that Gpr18 and Cbr1 are likely not required for this response.

### 3.4. Endocannabinoids Can Alter Baseline and PTZ-Induced Activity in Zebrafish Larvae

**Figure 4:**
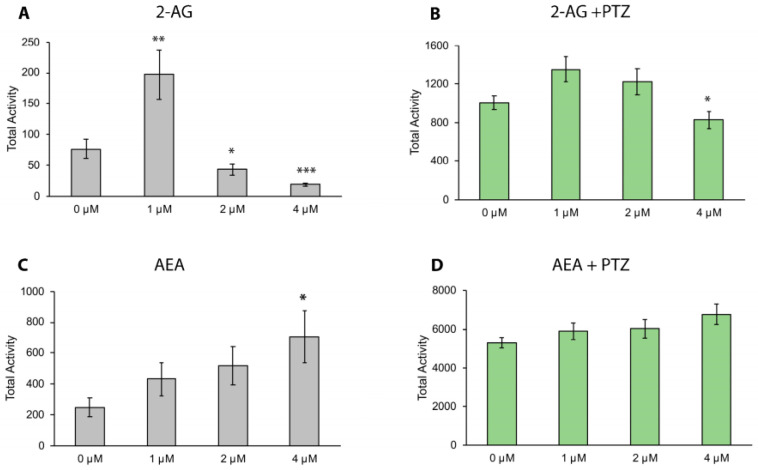
The endocannabinoid 2-AG alters baseline movement and PTZ-induced hyperactivity. Movement assayed before the addition of PTZ demonstrates increased activity at the 1 μM dose for 2-AG and reduced movement at 2 and 4 μM doses (**A**). The 4 μM dose of AEA significantly increased baseline movement (**C**). After the addition of PTZ, reduced movement is observed compared to controls at the 4 μM dose of 2-AG (**B**), with no effect observed for AEA (**D**). Data presented as mean +/− SEM, * *p* < 0.05, ** *p* < 0.01, *** *p* < 0.001 (compared to 0 μM). n = 40–48 larvae per condition.

Given the proposed overlap in the mechanism of function between endocannabinoids and phytocannabinoids, the potential of treating seizures with endocannabinoids was investigated. In larvae not exposed to PTZ, the addition of 2-AG increased activity when applied at a 1 μM dose (Figure 4A), yet showed evidence of a sedative effect at the 2 and 4 μM doses (Figure 4A). While a trend of increased activity was observed at all doses of AEA, only the 4 μM dose was statistically significant (Figure 4C). After addition of PTZ, a statistically significant reduction in hyperactivity was observed at the 4 μM dose of 2-AG (Figure 4B); however, no differences were observed for larvae pre-incubated in AEA (Figure 4D).

### 3.5. Phytocannabinoids Induce Changes in Gene Expression

**Figure 5:**
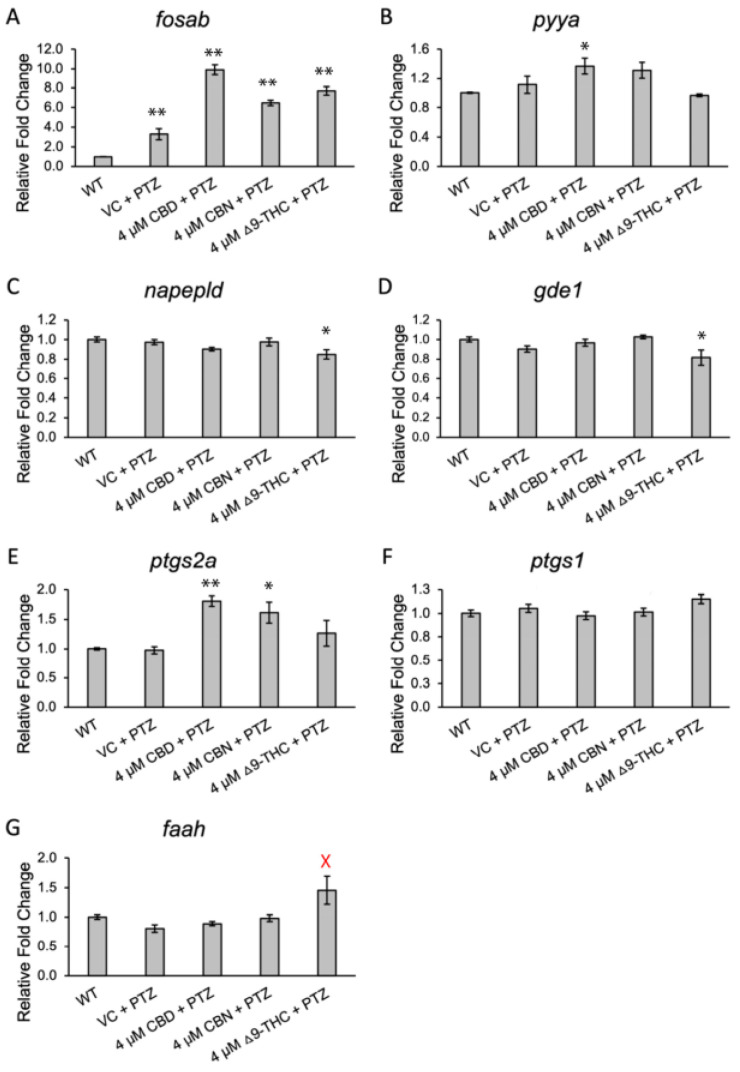
qPCR expression analysis of seizure markers and endocannabinoid system genes. (**A**–**G**) Methanol was used as the vehicle control, with 4 µM cannabinoid doses of cannabinoids used. Significance is noted via one-factor ANOVA with Tukey HSD, * *p* < 0.05, ** *p* < 0.01 (compared to WT). Red (X) indicates significance from PTZ/VC-treated larvae (*p* < 0.05). Error bars are SEM.

Based on the observed anticonvulsant properties, changes in the expression of neural and endocannabinoid pathway genes were evaluated in our seizure model after treatment with CBD, CBN, and Δ^9^-THC (Figure 5). Using qPCR on whole-body RNA, seven gene targets were investigated: *fosab*, *pyya*, *napepld*, *faah*, *gde1*, *ptgs2a*, and *ptgs1*. Expression of *fosab* was increased 3.3-fold (*p* < 0.01) via treatment with PTZ, as previously shown [8,49], and was further increased when combined with phytocannabinoid treatments (Figure 5A). Pre-incubation of PTZ-treated embryos with CBD resulted in a 9.9 ± 0.5-fold induction (*p* < 0.01), pre-incubation in CBN resulted in 6.5 ± 0.3-fold induction (*p* < 0.01), and Δ^9^-THC resulted in a 7.7 ± 0.4-fold induction (*p* < 0.01). While PTZ exposure did not affect the expression of *pyya,* it was significantly increased 1.4 ± 0.1-fold with combined PTZ and CBD treatment when compared to wild-type siblings (*p*< 0.05, Figure 5B). Expression of *napepld* was reduced in combined PTZ/Δ^9^-THC-treated embryos compared to WT larvae (0.8 ± 0.01-fold, *p* < 0.05, Figure 5C). Similarly, Δ^9^-THC was the only treatment to significantly decrease expression of *gde1* (0.8 ± 0.5-fold, *p* < 0.05, Figure 5D) when combined with PTZ. Expression of *ptgs2a* was unchanged after treatment with PTZ alone, but increased 1.8 ± 0.1-fold with CBD and PTZ treatment (*p* < 0.01) and 1.6 ± 0.2-fold with combined PTZ and CBN treatment (*p* < 0.05, Figure 5E). Expression of *ptgs1* was not significantly different in any treatment as compared to wild-type expression (Figure 5F). Expression of *faah* was increased after treatment with Δ^9^-THC as compared to the PTZ-treated embryos (1.5 ± 0.2-fold, *p* < 0.01), but not compared to WT siblings (Figure 5G).

## 4. Discussion

In this work, phytocannabinoids displayed potent anticonvulsant effects, similar to observations from previous studies [25,31]. The use of a novel HPLC method allowing for direct quantitation of phytocannabinoids in larvae builds upon previous studies [50,51], allowing for the analysis of activity per mass of cannabinoid that accumulates in larval tissues and demonstrates increasing larval accumulation with an increasing dose. The method runtime of 12 min is suitable for medium-throughput analysis, with separation of six phytocannabinoids.

Our seizure tracking data were consistent with a breadth of previous literature supporting the use of CBD as one of the most effective phytocannabinoids in the reduction of seizures. At a dose of 2 μM, the reduction of PTZ-based movement is likely a true anticonvulsant effect, as opposed to a sedative effect that has been previously described for some phytocannabinoids [31], as no statistically significant change in movement was observed before the addition of PTZ. CBN and CBC also showed a potent reduction of hyperactivity at dosages ranging from 1 to 4 µM and, interestingly, accumulated in larvae at similar levels. However, the 1 µM dosage showed reduced movement before the addition of PTZ, and, thus, the ability of these cannabinoids to reduce hyperactivity at the 1 µM dose is likely a combination of a sedative and an anticonvulsant effect. Like CBD, CBN and CBC showed no significant changes in baseline movement at the 2 μM dose, and, thus, the reduction of PTZ-induced hyperactivity likely represents a true anticonvulsant effect. Δ^9^-THC and its structural isomer Δ^8^-THC only provided seizure rescue at the highest concentrations used in this study (4 μM) and required higher accumulation within larvae for such effects. There was no evidence of sedation at this dose, as movement in non-PTZ treated larvae was increased at 4 µM, indicating the strong effect on PTZ-induced hyperactivity. It is unclear why high doses of THC and other phytocannabinoids increased movement in non-PTZ-treated larvae; however, previous reports have indicated high levels of cannabis extract may induce stress-related activity [52]. Interestingly, a 2 µM dose of CBG repeatedly increased movement to a level significantly higher than that of PTZ-treated larvae, while the 4 µM dose reduced activity. CBG has a broad range of binding affinities for a unique range of receptors, which has been reported to change based on concentration [53], which is likely the reason behind this unexpected dose–response trend.

After establishing the effects of treatment with phytocannabinoids, we sought to further investigate the mechanism of action of CBD-mediated seizure relief. By treating larvae with CBD, as well as antagonists of Cbr1, Gpr55, and Gpr18, a potential mechanism of action was determined by observing which conditions negatively affected seizure relief. These data show that CBD acts through Gpr55 as one modality to reduce seizures. When Gpr55 is chemically antagonized, CBD-induced seizure relief is significantly reduced, but not eliminated, suggesting there is more than one mechanism of action. When both Gpr55 and Cbr1 are blocked, the reduction in seizure relief is slightly more than the inhibition of Gpr55 alone, but this was not statistically significant. While other reports demonstrate that inhibition of Cbr1 with AM251 affects the ability of CBD to reduce baseline movement [54], our study suggests that Cbr1 may play a minor role in mediating CBD’s anticonvulsant effects. A number of studies have also suggested that phytocannabinoids can bind to Gpr18 [55,56]; however, blocking Gpr18 showed no effect on CBD-induced seizure reduction and thus this cannabinoid receptor likely does not transduce the anticonvulsant effects of CBD. Notably, none of the receptor antagonists significantly altered movement in larvae before addition to PTZ, and, thus, the seizure relief afforded by CBD (or lack thereof via co-incubation with CBD and the Gpr55 antagonist ML-193) is likely attributable to the inability of CBD to bind the receptor in question, as opposed to potential changes in movement due to the chemical antagonist itself.

Given the role of the endocannabinoid receptors in mediating the anticonvulsant effects of phytocannabinoids, we sought to better understand the potential role of endocannabinoid ligands in the production of, or relief from, seizures. Endocannabinoid ligands and their resulting signaling pathways are generally considered protective with respect to seizures [44,57]; however, there are conflicting data. For example, while AEA has been shown to have anticonvulsant effects, loss of the enzyme FAAH that catabolizes AEA to arachidonic acid (AA) renders AEA treatment proconvulsive [58,59]. This suggests that AA may actually mediate the anticonvulsant effects observed in AEA treatment, or that the ratio of AEA to AA may be important in regulating neuronal hyperexcitability. In zebrafish, mutation of *faah* genes affects locomotor activity in response to stress [60], further highlighting the role of this gene in regulating movement and hyperactivity. As changes in endocannabinoid ligand levels have been noted in cannabis users, we hypothesized that the induction of seizures, or their relief through phytocannabinoid exposure, may alter endocannabinoid ligand synthesis. The expression of *napepld*, *gde1* (synthesis of AEA), and *faah* (breakdown of AEA) was not significantly affected by PTZ treatment, which suggests that they are not involved in seizure induction and progression in this model. There was, however, a significant reduction of *napepld* and *gde1* expression after Δ^9^-THC exposure in PTZ-treated embryos. As AEA and Δ^9^-THC have been reported to act on the same receptors [30,37,61], these data may indicate a negative feedback loop whereby binding of Δ^9^-THC to endocannabinoid receptors could inhibit production of their endogenous ligands. Expression of *faah,* required for the breakdown of AEA to AA, was significantly increased in PTZ-exposed embryos pre-treated with Δ^9^-THC when compared to PTZ treatment only. Together, these results suggest that Δ^9^-THC may change the availability of AEA in PTZ seizure models or alter the ratio of AEA to AA; however, this needs to be tested directly.

Expression of the zebrafish ortholog of *cox-2* (*ptgs2a*) was increased after phytocannabinoid treatment when compared to wild-type embryos, with results from CBD and CBN treatment being statistically significant. COX-2 is responsible for the oxidation of a minor amount of 2-AG and AEA [37,62], and, thus, endocannabinoid levels may also be reduced after CBD or CBN treatment, although this was not directly tested. As we have demonstrated that incubation of larvae in endocannabinoids can alter baseline and PTZ-induced hyperactivity in our model, changes in endocannabinoid synthesis could potentially represent a component of the anticonvulsant effects of Δ^9^-THC, CBD, and CBN.

Lastly, we assessed known neuronal markers of seizure activity to determine whether their alteration by phytocannabinoids represents a path toward seizure relief. The neuronal activity gene c-Fos (*fosab*) commonly serves as a marker for seizure progression in animal model studies [8,63]. Some ASMs have been shown to decrease *fosab* expression to wild-type levels [12]. While incubation of larvae in PTZ more than doubled the expression of *fosab*, we found that treatments of CBD, CBN, and Δ^9^-THC dramatically increased *fosab* expression further, even though larvae in these treatment groups displayed reduced seizure activity. Treatment with CBD or Δ^9^-THC was reported to cause a dose-dependent increase in *fosab* expression in 96 hpf embryos [51], and a fear-learning study found that only specific brain structures increase *fosab* expression following Δ^9^-THC treatment [34]. While these data do not support a model of *fosab* as a marker of seizure progression, they do support a hypothesis whereby larvae may increase expression of *fosab* after seizures as a compensatory mechanism, and the further increase with phytocannabinoid treatment could, thus, account for the therapeutic benefit observed.

Previous work has demonstrated that seizures induced expression of the signaling peptide encoded by *pyya* [12,64]. In our study, there was no statistically significant increase of *pyya* expression after PTZ treatment; however, the PTZ dose and the age of larvae used differ among the studies. The expression of *pyya* is increased in larvae treated with CBD and PTZ exposure compared to wild-type larvae, indicating that CBD and PTZ may be activating similar pathways toward *pyya* regulation. Some studies have associated peptide YY with the inhibition of seizures and neuroprotection from stress [65,66], and, thus, its increase after combined CBD and PTZ exposure, but not with PTZ alone, may indicate a mechanism for seizure relief.

In conclusion, a robust and high-throughput behavioral analysis method yielded sensitive and accurate measurements of hyperactivity reduction resulting from phytocannabinoid treatment in a well-studied seizure model. A novel HPLC method was employed for the sensitive and reliable measurement of six therapeutically important phytocannabinoids in pooled zebrafish larvae. CBD, CBC, and CBN were observed to have significant anticonvulsant effects at low doses, with CBC and CBN accumulating at lower tissue concentrations to provide such effects. CBD-induced seizure relief is partially mediated by Gpr55, and our assays can now be repeated to assess the roles of these receptors in mediating the anticonvulsant effects of additional phytocannabinoids, such as CBC and CBN. The increased expression of *fosab* during seizures is further increased with phytocannabinoid treatment, suggesting that increasing *fosab* expression may be a compensatory mechanism. The role of the endocannabinoid system in phytocannabinoid seizure relief, including genes involved in the synthesis and breakdown of AEA and 2-AG and the Gpr55 receptor, is supported by this study.

## Data Availability

The principal Investigator (C.R.F.) can be contacted for access to any data pertaining to this manuscript.

## Supplementary Materials

The following supporting information can be downloaded at: https://www.mdpi.com/article/10.3390/biom13091398/s1, Figure S1: Phytocannabinoid extraction chromatograms; Figure S2: External calibration curves across the linear range of each compound, presented as average of three different days; Table S1: Cannabinoid retention times, quantification wavelengths and analytical figures of merit of the presented method.
