# Supplementary material for: Phytocannabinoids Reduce Seizures in Larval Zebrafish and Affect Endocannabinoid Gene Expression
Source: Biomolecules. 2023 Sep 16;13(9):1398. doi: 10.3390/biom13091398 (PMC10526363; doi:10.3390/biom13091398)
Supplement: Supplementary file 1 [file biomolecules-13-01398-s001.zip › biomolecules-2544839-supplementary.pdf]

Supplementary Information

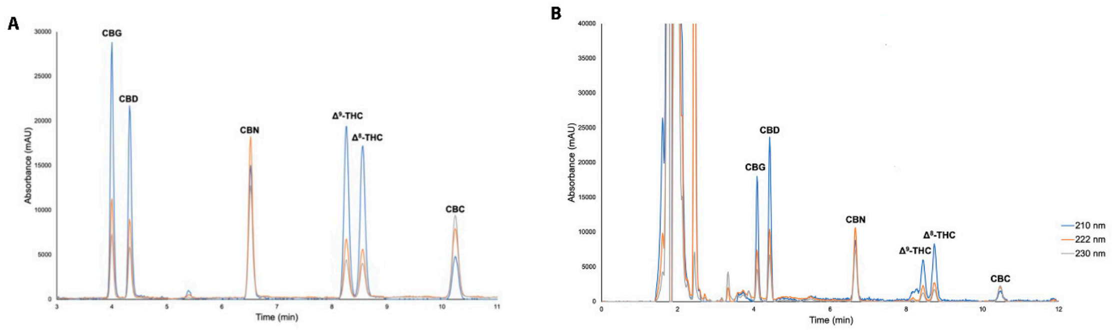

Figure S1: Phytocannabinoid extraction chromatograms. Representative chromatogram for a mixed standard of cannabinoids (2.5 µg/ml) in extraction solvent at three wavelengths (A). Representative chromatogram of an extraction from 12 pooled larvae treated with 6 phytocannabinoids at a concentration of 4 µM (B).

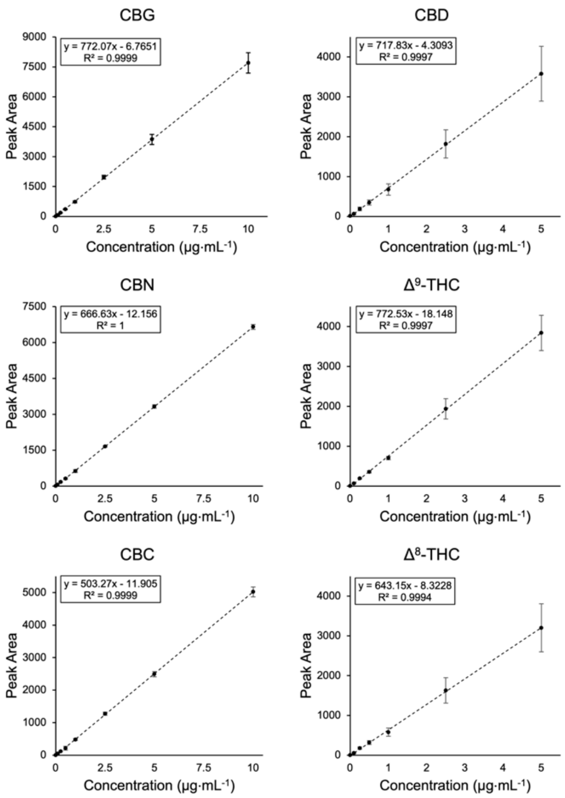

Figure S2: External calibration curves across the linear range of each compound, presented as average of three different days. Error bars represent the standard deviation between the

days. Linear regression analysis is plotted as dotted lines with the equation for the line of best fit and the linear correlation coefficient (R^2) in the overlaid boxes.

Table S1. Cannabinoid retention times, quantification wavelengths and analytical figures of merit of the presented method. RT = retention time; Quant. WL = quantification wavelength; LLOD = lower limit of detection; LLOQ = lower limit of quantification; IDV = inter-day variability.

**Analyte**	**RT (min)**	**Quant. WL (nm)**	**LLOD ($\mu g \cdot mL^{-1}$ for 20 µL inj.)**	**LLOQ ($\mu g \cdot mL^{-1}$ for 20 µL inj.)**	**LLOD ng/larva**	**LLOQ ng/larva**	**Linear range and R^2, n=3**	**IDV (%, n=10)**
CBG	4.0	210	0.02	0.07	0.27	1.01	0.1-10<br>0.9999	5.6
CBD	4.3	210	0.02	0.08	0.28	1.13	0.1-5<br>0.9997	7.7
CBN	6.6	222	0.01	0.05	0.17	0.66	0.1-10<br>1.00	3.1
Δ^9-THC	8.3	210	0.01	0.08	0.12	1.04	0.1-5<br>0.9997	5.9
Δ^8-THC	8.6	210	0.01	0.10	0.17	1.45	0.1-5<br>0.9994	8.6
CBC	10.3	230	0.03	0.08	0.35	1.11	0.1-10<br>0.9999	1.3
